# Ectopic Adrenocorticotropic Hormone (ACTH) Syndrome Caused by Mediastinal Carcinoma: A Report of a Rare Case

**DOI:** 10.7759/cureus.102195

**Published:** 2026-01-24

**Authors:** Sara Hassane, Zineb Serhane, Mohammed Amine Essafi, Zineb Elazime, Hayat Aynaou, Houda Salhi

**Affiliations:** 1 Department of Endocrinology, Diabetology, Metabolic Diseases and Nutrition, Hassan II University Hospital, Fez, MAR

**Keywords:** acth, cushing's syndrome, ectopic, mediastinal carcinoma, neuroendocrine tumor

## Abstract

Ectopic adrenocorticotropic hormone (ACTH) syndrome (EAS) is a rare cause of ACTH-dependent Cushing's syndrome, exceptionally due to mediastinal carcinoma. We report a 37-year-old man with Cushingoid features, hypercortisolism, and elevated ACTH. Imaging revealed an unresectable anterior mediastinal mass; biopsy confirmed high-grade neuroendocrine carcinoma. Despite medical therapy and chemoradiotherapy, the outcome was fatal within months. This case highlights the rarity, diagnostic difficulty, and poor prognosis of mediastinal EAS.

## Introduction

Cushing's syndrome refers to a group of disorders caused by excessive cortisol secretion, with an estimated incidence ranging from 1.8 to 3.2 cases per million per year. In adrenocorticotropic hormone (ACTH)-dependent forms, the majority of cases are related to pituitary adenomas, whereas ectopic ACTH syndrome (EAS) accounts for approximately 9-18% of cases [1]. However, the reported prevalence of EAS varies considerably across studies, reflecting differences in study populations, diagnostic criteria, and referral center bias.

EAS results from non-pituitary tumors producing ACTH, most commonly small cell lung carcinoma, thymic carcinoma, or pancreatic neuroendocrine tumors (NETs). Thymic NETs are rare, representing approximately 2% of mediastinal masses. Among functional thymic NETs, ACTH secretion is reported in 40-50% of cases, yet they constitute only a small proportion of all EAS etiologies [2].

Isolated reports have described mediastinal ectopic ACTH sources, including typical carcinoid tumors, paragangliomas, and exceptionally aggressive thymic NETs. These entities are exceedingly rare and often pose significant diagnostic and therapeutic challenges due to their atypical mediastinal location, their rapid clinical progression, and the frequent need for urgent multidisciplinary management [3].

We report a rare case of ACTH-dependent Cushing's syndrome secondary to a mediastinal carcinoma, distinguished by its aggressive histological features and rapid disease progression, thereby highlighting the complexity of diagnosing and managing ectopic ACTH secretion from mediastinal malignancies.

## Case presentation

**Figure 1:**
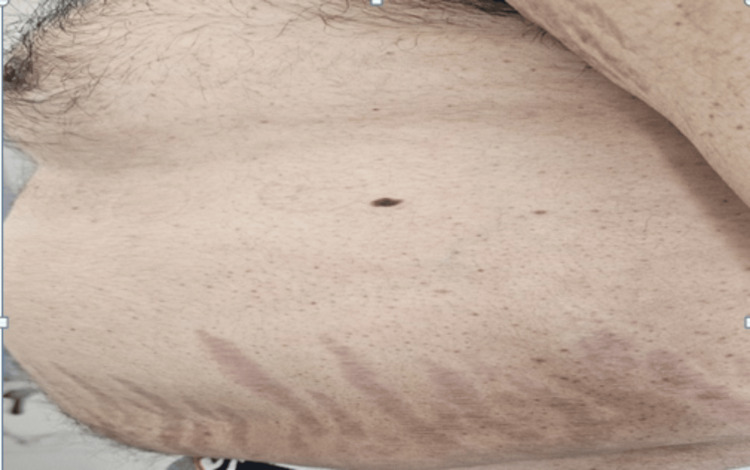
Clinical images of the patient showing Cushingoid features

A 37-year-old man was admitted to our Endocrinology Unit. The patient presented with proximal muscle weakness and progressive weight gain. He had central obesity (body mass index (BMI) 28 kg/m², waist circumference 110 cm) with characteristic Cushingoid features: moon face, dorsocervical fat pad, and abdominal striae (Figure 1).

Biochemical evaluation revealed markedly elevated 24-hour urinary free cortisol (UFC) of 630 μg/24 hours (reference range: 20-90 μg/24 hours) and morning serum cortisol of 38 μg/dL (reference range: 6-18 μg/dL). ACTH levels were 95.7 pg/mL (reference range: 10-60 pg/mL), confirming ACTH-dependent Cushing's syndrome. Loss of circadian cortisol rhythm and lack of suppression after a low-dose dexamethasone test were also noted.

Additional laboratory findings included hyperglycemia, leukocytosis with relative lymphopenia, and normal potassium levels (Table 1).

**Table 1:** Biological results at admission ACTH: adrenocorticotropic hormone

Laboratory parameter	Result	Reference range	Units
24-hour urinary free cortisol	Elevated >3 × the upper limit of normal	20-90	µg/24 hours
Morning plasma cortisol (8:00 a.m.)	Loss of circadian rhythm (elevated)	-	µg/dL
Midnight plasma cortisol	Elevated	<7.5	µg/dL
Low-dose dexamethasone suppression test (1 mg): post-test cortisol	24 (not suppressed)	<1.8	µg/dL
Plasma ACTH	95.7	10-60	pg/mL
Fasting plasma glucose	Elevated	70-100	mg/dL
White blood cell count	Elevated (leukocytosis)	4,000-10,000	/mm³
Lymphocyte count	Reduced (relative lymphopenia)	1,000-4,800	/mm³
Serum potassium	Normal	3.5-5.1	mmol/L
Plasma metanephrines	Normal	<90 (normetanephrine), <180 (metanephrine)	pg/mL

**Figure 2:**
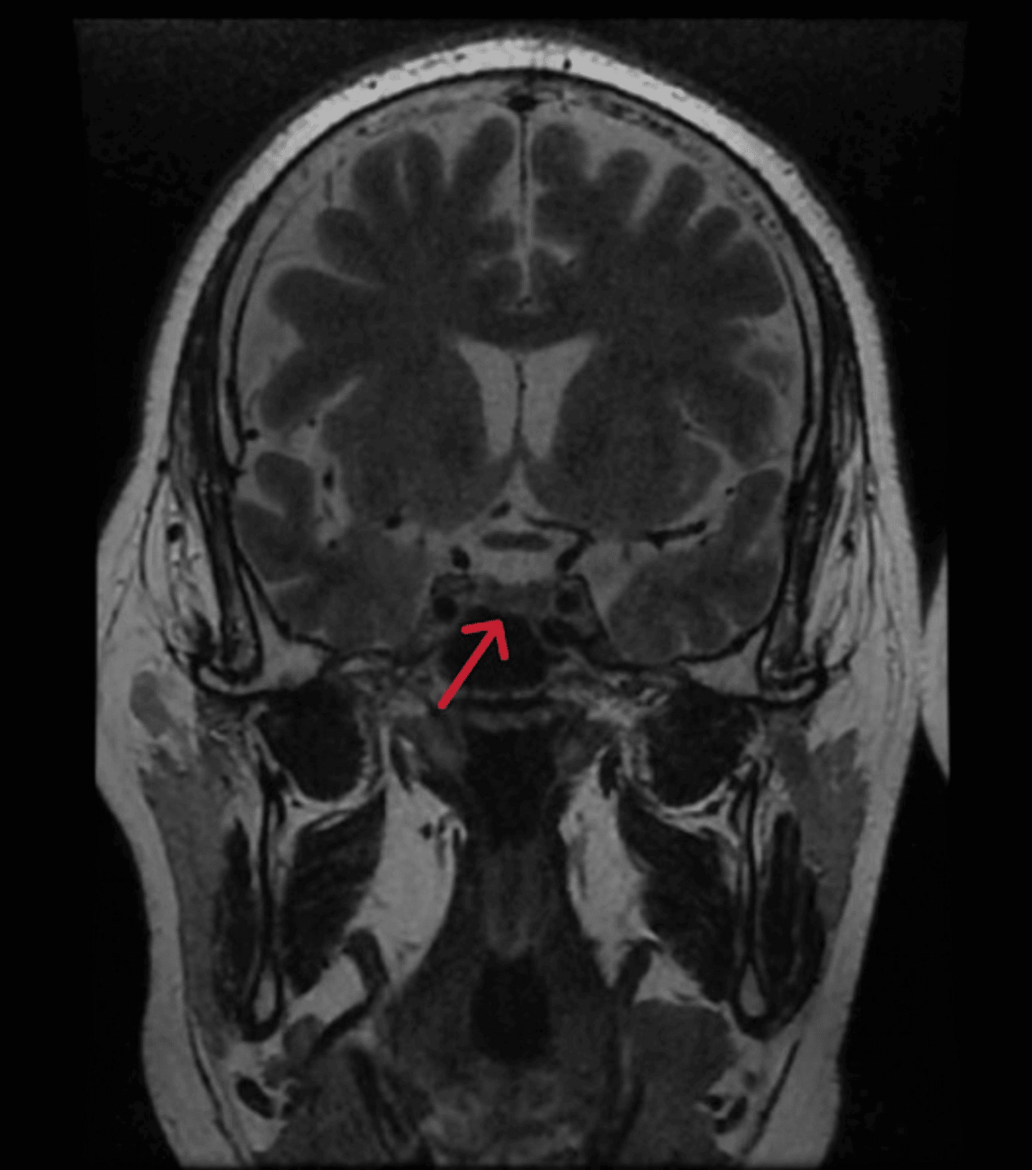
Image showing a pituitary gland of normal size, shape, and signal intensity, with no evidence of adenoma (arrow)

Inferior petrosal sinus sampling (IPSS) was not performed due to a lack of availability. Nevertheless, biochemical and imaging findings strongly suggested an ectopic source of ACTH. Pituitary MRI was normal, with no evidence of micro- or macroadenoma (Figure 2).

**Figure 3:**
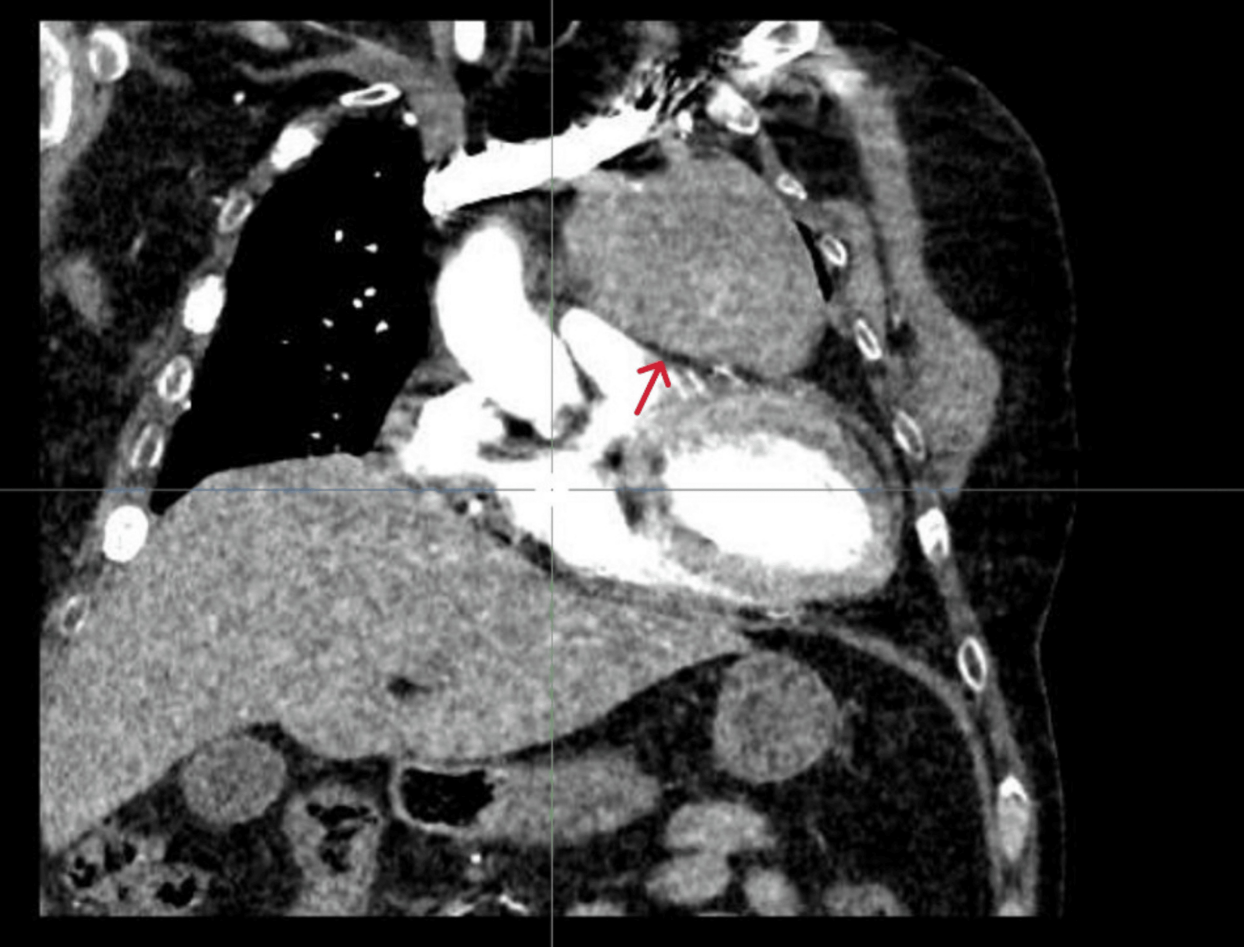
Coronal contrast-enhanced CT scan of the chest showing a large heterogeneous mediastinal mass extending towards the carina and involving adjacent mediastinal structures, including close contact with major vessels such as the ascending aorta and pulmonary artery. This suggests a locally advanced, unresectable tumor, likely of bronchopulmonary or lymphomatous origin (arrow)

A thoraco-abdominopelvic CT scan revealed a large anterior mediastinal mass, measuring 9 × 6.3 × 7 cm. It had irregular contours, was heterogeneous, and showed punctate calcifications and areas of necrosis. These findings were suggestive of thymic carcinoma (Figure 3). Further evaluation with thoracic MRI and CT angiography confirmed a hypervascular mass. Plasma metanephrines were normal, ruling out a functional paraganglioma.

Extrathoracic metastases were assessed using thoraco-abdominopelvic CT, and no distant lesions were detected.

**Figure 4:**
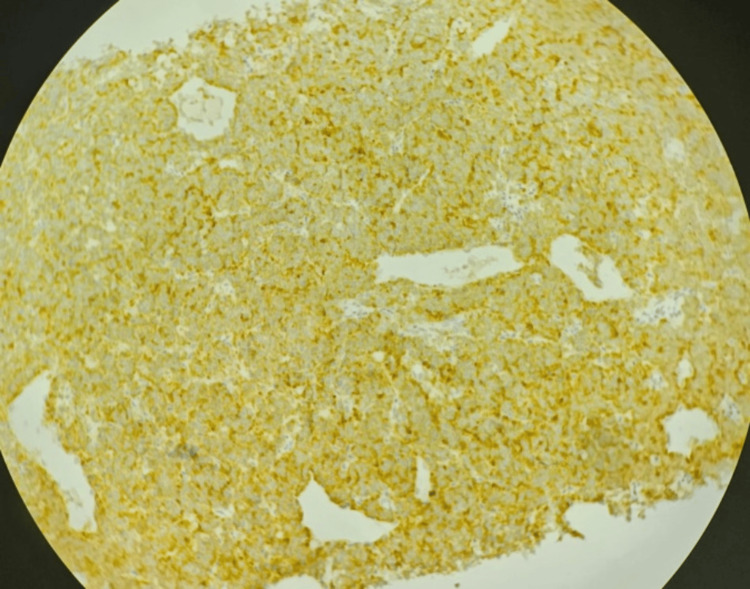
Immunohistochemical staining for synaptophysin in mediastinal tumor tissue demonstrating strong cytoplasmic positivity. Counterstained with hematoxylin. Original magnification: ×200

**Figure 5:**
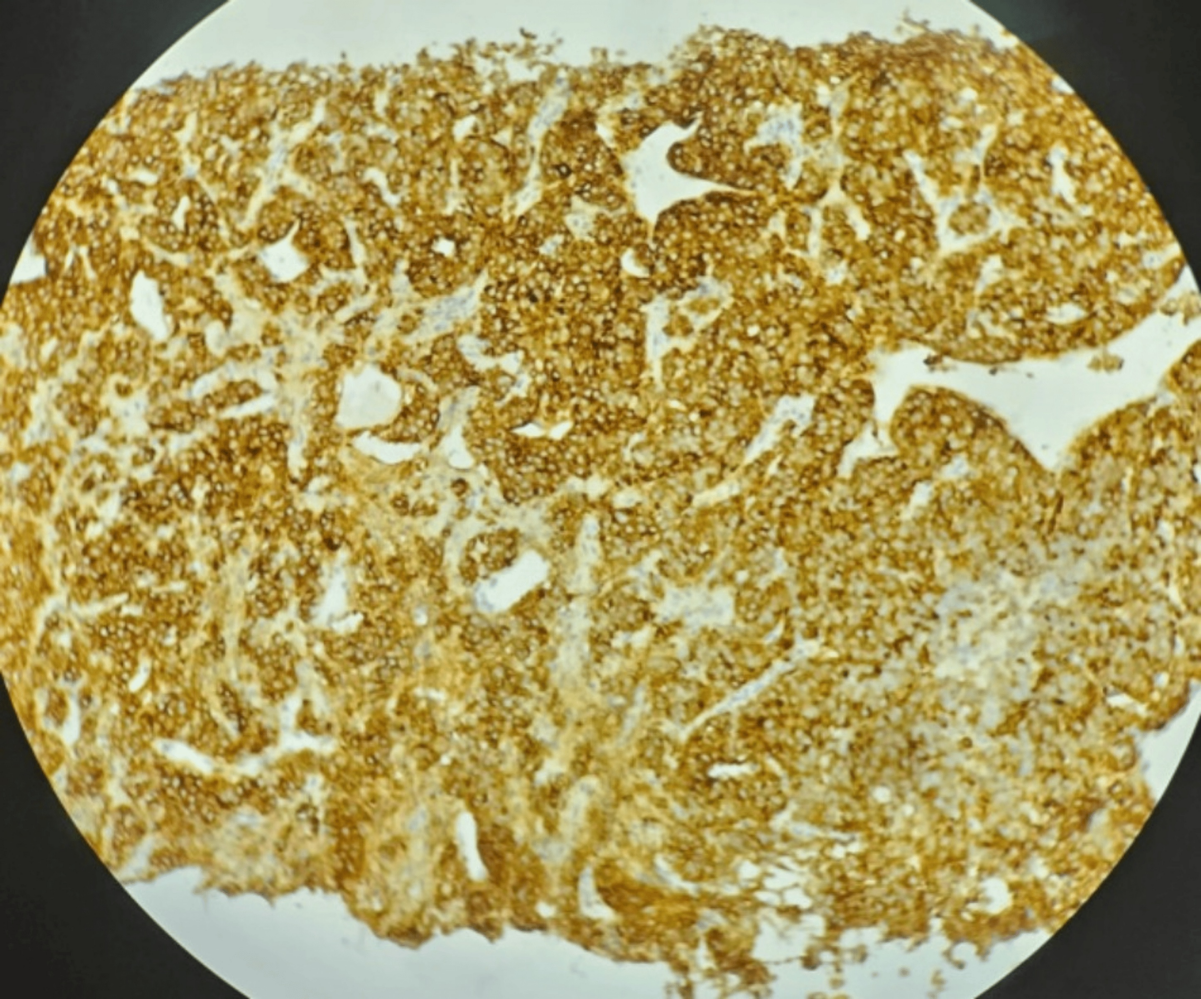
Immunohistochemical staining for chromogranin A in mediastinal tumor tissue demonstrating robust granular cytoplasmic positivity consistent with neuroendocrine tumor. Counterstained with hematoxylin. Original magnification: ×400

A CT-guided biopsy was performed. Immunohistochemistry was positive for chromogranin A, synaptophysin, and cytokeratin. The Ki-67 index was 67% in the most proliferative areas, consistent with a high-grade neuroendocrine carcinoma (NET G3) according to the World Health Organization (WHO) classification. Paraganglioma was excluded based on cytokeratin positivity, and lymphoma and germ cell tumors were ruled out based on morphological and immunohistochemical features (Figure 4 and Figure 5).

The case was discussed in a multidisciplinary team meeting. The tumor was deemed inoperable due to extension towards the carina and adjacent structures. The patient was referred for palliative chemoradiotherapy.

Despite early diagnosis and treatment initiation, the clinical course was unfavorable. The patient's condition worsened progressively, and he died a few months later due to cardiac decompensation.

## Discussion

Cushing's syndrome results from prolonged exposure to elevated glucocorticoid levels. When ACTH is elevated, the condition is classified as ACTH-dependent, with the most common cause being pituitary adenomas (Cushing's disease). However, in approximately 10-15% of ACTH-dependent cases, the source is ectopic, most frequently small cell lung carcinoma and thymic NETs [1]. These ectopic tumors bypass normal hypothalamic-pituitary regulation, secreting ACTH autonomously and inducing bilateral adrenal hyperplasia and excess cortisol production, leading to the typical clinical features of Cushing's syndrome: central obesity, facial rounding, muscle weakness, hypertension, glucose intolerance, and hypokalemia [4].

In our case, the patient presented with classic signs of hypercortisolism: moon face, buffalo hump, proximal muscle wasting, and severe metabolic disturbances, including poorly controlled diabetes. Potassium levels remained normal throughout. Biochemistry confirmed ACTH-dependent hypercortisolism, with non-suppressed cortisol after the dexamethasone test, and markedly elevated ACTH. Given these findings, the next critical step was to localize the ACTH-secreting tumor.

Imaging studies (CT of the thorax) revealed a mediastinal mass, a rare but reported source of ectopic ACTH secretion. Mediastinal carcinomas, including thymic and poorly differentiated NETs, represent uncommon etiologies of EAS and often present with non-specific symptoms such as chronic cough, chest pain, or weight loss, which were also noted in our patient.

Histological analysis confirmed a high-grade mediastinal carcinoma with neuroendocrine features, confirming the diagnosis of EAS [5].

Surgical resection, the preferred treatment when feasible, was ruled out due to vascular invasion and mediastinal encasement, confirmed by thoracic surgery and imaging teams. Consequently, the patient was managed with medical cortisol-lowering therapy (ketoconazole) and referred for combined radio-chemotherapy, which is the standard approach for inoperable neuroendocrine carcinomas [6].

EAS carries a poor prognosis, particularly when the tumor is unresectable. In such cases, long-term control of hypercortisolism is essential to reduce morbidity and mortality. Literature reports suggest that only 47% of patients achieve remission, even after tumor resection, and that long-term follow-up is required due to the risk of recurrence, particularly in NETs [7,8].

Despite the early diagnosis and initiation of appropriate therapy, the clinical course remained unfavorable. The patient's condition progressively deteriorated, and he died a few months later as a result of cardiac decompensation. This outcome highlights the aggressive nature of ectopic ACTH-secreting mediastinal tumors and underscores the importance of early localization, multidisciplinary management, and close monitoring, even when curative surgery is not feasible [9,10].

## Conclusions

EAS remains a diagnostic challenge due to its often subtle or non-specific presentation and the difficulty in localizing the source. It is frequently underrecognized and misdiagnosed. When feasible, surgical resection of the ACTH-secreting tumor is the treatment of choice, offering the best chance for complete clinical remission.

This case highlights the importance of considering mediastinal carcinoma in the differential diagnosis of EAS. Functional imaging is essential for tumor localization, and timely intervention remains critical to improving outcomes.

## References

[1] Lacroix A, Feelders RA, Stratakis CA, Nieman LK (2015). Cushing's syndrome. Lancet.

[2] Lawrence L, Zhang P, Choi H, Ahmad U, Arrossi V, Purysko A, Makin V (2019). A unique case of ectopic Cushing's syndrome from a thymic neuroendocrine carcinoma. Endocrinol Diabetes Metab Case Rep.

[3] Li B, Yan Z, Huang H (2021). Case report: an unusual case of ectopic ACTH syndrome caused by mediastinal paraganglioma. Front Endocrinol (Lausanne).

[4] Witek P, Witek J, Zieliński G, Podgajny Z, Kamiński G (2015). Ectopic Cushing's syndrome in light of modern diagnostic techniques and treatment options. Neuro Endocrinol Lett.

[5] Dzialach L, Wojciechowska-Luzniak A, Maksymowicz M, Witek P (2024). Case report: a challenging case of severe Cushing's syndrome in the course of metastatic thymic neuroendocrine carcinoma with a synchronous adrenal tumor. Front Endocrinol (Lausanne).

[6] Isidori AM, Sbardella E, Zatelli MC, Boschetti M, Vitale G, Colao A, Pivonello R (2015). Conventional and nuclear medicine imaging in ectopic Cushing's syndrome: a systematic review. J Clin Endocrinol Metab.

[7] Elenius H, Nieman LK (2025). Recognition and management of ectopic ACTH secreting tumors. J Endocr Soc.

[8] Sharma ST, Nieman LK, Feelders RA (2015). Cushing's syndrome: epidemiology and developments in disease management. Clin Epidemiol.

[9] Jia R, Sulentic P, Xu JM, Grossman AB (2017). Thymic neuroendocrine neoplasms: biological behaviour and therapy. Neuroendocrinology.

[10] Newell-Price J, Bertagna X, Grossman AB, Nieman LK (2006). Cushing's syndrome. Lancet.

